# Consequences of obstetric fistula in sub Sahara African countries, from patients’ perspective: a systematic review of qualitative studies

**DOI:** 10.1186/s12905-018-0605-1

**Published:** 2018-06-20

**Authors:** Debrework Tesgera Bashah, Abebaw Gebeyehu Worku, Mezgebu Yitayal Mengistu

**Affiliations:** 1 School of Nursing, College of Medicine and Health Sciences, University of Gondar, P. O. Box 196, Gondar, Ethiopia; 2 Department of Reproductive Health, Institute of Public Health, College of Medicine and Health Sciences, University of Gondar, Gondar, Ethiopia; 3 Amhara National Regional State Health Bureau, Bahir Dar, Ethiopia; 4 Department of Health Service Management and Health Economics, Institute of Public Health, College of Medicine and Health Sciences, University of Gondar, Gondar, Ethiopia

**Keywords:** Challenges of incontinence, Obstetric fistula, Psychosocial experiences, Urogenital fistula, Vesicovaginal fistula

## Abstract

**Background:**

Women with fistula live in a state of distress and in fear of their future life. An obstetric fistula has a devastating impact on affected women and their families. The objective of this systematic review was to synthesize the evidence from published articles on the consequences of obstetric fistula on women who endure the condition.

**Methods:**

The consequences were systematically reviewed from purely qualitative and mixed method primary studies. The literatures were searched through the search engines Google, Google scholar, Hinari using Pub Med data bases, and citation tracking. Relevant source of publications were searched for primary qualitative studies by formulating search protocol using related search terms. Time (articles published between January first of 2007 and 30th September 2016), participants (women who experienced obstetric fistula due to obstructed labor complications), types of study (purely qualitative and mixed method primary articles), findings (reporting consequences/impacts of obstetric fistula) were used as inclusion criteria. The quality appraisal tool for qualitative studies and the critical appraisal skills program were used to appraise the quality of the studies. The findings of sixteen studies were included in the review. The data were collected and then a thematic framework approach was applied for analysis.

**Results:**

The thematic categories shared across most studies were related to the physical challenges of losing body control, women’s social and family relationships, and the challenges of losing income. Obstetric fistula has far reaching consequences on women’s physical well being, social and marital relationships, mental health and economic capacity. Fistula also challenged women coping abilities.

**Conclusion:**

The consequences of obstetric fistula are far more than the visible medical condition. Little evidence is available on mental health, child and fertility issues, and coping mechanisms. Therefore, further researches shall be aimed at addressing the understudied area and suitable interventions shall be offered to improve women’s overall quality of life.

**Electronic supplementary material:**

The online version of this article (10.1186/s12905-018-0605-1) contains supplementary material, which is available to authorized users.

## Background

Obstetric fistula is a preventable maternal morbidity that results from prolonged and obstructed labor [1]. It commonly occurs when there is Cephalo pelvic disproportion. Unless there is skilled obstetric intervention this disproportion creates pressure on the tissues and then prolonged ischemia will cause tissue necrosis leading to fistula formation [2]. It causes life-long disabilities and poor quality of life [3, 4]. In the developed world it is very rare and faded away 100 years ago following improved obstetric care [5], while it remains the prevalent cause of maternal morbidity in the developing world [6, 7]. It affects more than 2 million women worldwide, with at least 50,000 to 100,000 new cases occurring annually [8]. Among which the majority is from resource-poor countries where the health system is ineffective [9, 10]. The majority of obstetric fistula cases are in Africa and Asia [1]. Continuous and uncontrollable leaking of urine and/or feces can lead to life-changing stigmatization of the women [11, 12]. Therefore women with fistula live in a state of distress and a fear of their future life [13]. The consequence of obstetric fistula is devastating for those affected women and their families [14, 15]. Beyond the medical conditions, the social consequences are severe, and affected women are often ostracized from their community, divorced, abandoned, and remain childless [16, 17]. Women living with fistula may be blamed by the community members for their condition, viewing it as punishment for sin or a venereal disease or curse [17]. The women are unable to participate in religious activities and social gatherings, and are considered unhygienic [18, 19].

The objective of this systematic review was to summarize and synthesize the evidence of the multidimensional consequences on women with obstetric fistula. It can therefore provide evidence for decision makers for intervention so as to improve women’s quality of life.

## Methods

### Search strategy

The database searches used specified key terms to identify studies for potential inclusion in the review. The literature was searched through search engines Google, Google scholar, and Hinari, using Pub- Med data bases and citation tracking.

CoCoPopS protocol was formulated to identify articles Co: obstetric fistula impact/challenge; Co: developing world/sub-Saharan Africa; Pop: women with obstetric fistula; and S: primary qualitative and/or mixed method studies.

The terms were; “Obstetric fistula”,“vesicovaginal fistula”, “Rectovaginal fistula”, “Urogenital fistula”, “challenges of fistula”, “Consequences of fistula”, “Psychosocial experience” of women were key words used to search relevant papers and the search protocol was as follows:

“Obstetric fistula” [MeSH Terms] OR “Obstetric fistula” [All Fields] OR“VVF” [MeSH Terms] OR “Vesico-vaginal Fistula[MeSH Terms]OR” “Rectovaginal Fistula” [MeSH Terms] OR “RVF” [MeSH Terms] OR “Urogenital fistula” [MeSH Terms] AND “Consequence” [MeSH Terms] OR“Consequence” [All Fields] OR “Challenges” [All Fields] OR “Lived experience” [All Fields] AND “Africa” [All Fields] OR “sub-Sahara Africa” [All Fields]. There were no language restrictions except for articles which did not have the “translate” option. As a result, one article was found in French and was excluded due to the irrelevance of its objective with the review objective. Predominantly, the review was based on a selection of published literature. Three steps were used to select papers for review. First, relevant titles and abstracts were identified from databases. In the second stage, screening and retrieving of full text articles were conducted. In the final step, after identifying papers that potentially meet the inclusion criteria, data extraction from relevant selected articles and qualitative reviews of the articles were done. Analysis was restricted to studies intended to explore the consequences of obstetric fistula on women with the condition.

### Selection of studies

For studies that have an abstract identified in the database searches, the abstracts were reviewed to determine whether or not they should be included by using the following criteria for the inclusion and exclusion of papers.

#### Phase I

##### Topic:

An article was included only if it discussed obstetric fistula and its potential consequences/experiences/challenges on women with obstetric fistula.

##### Time:

Articles published between January 1^st^2007 and 30th September 2016 was included for the review.

##### Participants:

Study participants had to be women who experienced obstetric fistula Rectovaginal fistula (RVF) or Vesicovginal fistula (VVF) due to obstructed labor complications.

##### Type of study:

The primary articles of case studies, and purely qualitative and/mixed method studies, which used both qualitative and quantitative approach were included.

#### Phase II

Selected articles were read in their full document and included if only they reported the consequences/impacts of obstetric fistula regardless of respondents’ repair status (whether the study was conducted before or after repair). Articles were excluded if there was no discussion of the impact/ challenge/experiences of fistula victims.

For each article included in phase II, the article information was entered into a structured data extraction form. The information included in the data extraction form included the name of the author, publication date, country, setting, study design, participant characteristics, and consequences identified (Table 1). The identified quality appraisal framework Critical appraisal skills Program (CASP) was used for each study included [20]. Selected studies were assessed with the listed criteria in CASP, such as clarity of the objectives, appropriateness of method used, clarity of the study context, appropriateness of the participant recruitment strategy to the aims of the research, data collection technique, relationship between researcher and participant (bias), consideration of ethical issues, whether data analysis was sufficiently rigorous, whether findings were clear, how much the study was valuable regarding its transferability, and contribution to knowledge and identifying areas of research gap (Additional file 1). Each study has scored good to poor in each criterion, with a quality rating score of high to low with respective percentage values. A total of 16 studies that met the inclusion criteria were included in the review.

**Table 1:** Characteristics of sixteen qualitative / mixed studies reporting consequences of obstetric fistula in sub Saharan Africa

Author(s) year of publication	Location, setting	Study design	Focus of study (Objectives)	Methods	Sample Characteristics	Consequences
Jenny. H and Sanna. S, 2014	Tanzania Facility	NR	To learn about psychological consequences resulting from fistula.	Interviewer administered questionnaire	*n* = 63,mean age at fistula development =31,mean time with fistula = 8.8 year	Decreased ability/ or inability to work, borrowed money, saved for bus fair for 3 years Divorced/rejected Lost babies and remain childless isolation
Mselle et al., 2012	Tanzania Facility	Qualitative	Explore Women’s experience of fistula	Interview	*n* = 8 median age = 30 yrs. median years with fistula = 6	Uncertainty about being accepted
Muleta et al., 2008	Ethiopia (Rural)	Mixed method	To assess health, social and psychological problems encountered by women	In-depth interview	*n* = 13 median age = 33 age range at dev. of fistula = 15–49 median duration of labor = 4 days	amenorrhea, leg pain, difficulty of walking
Pope, Bangser and Requejo, 2011	Tanzania Facility	Mixed methods	Explore barriers and facilitating factors women experience reintegrating in to society after treatment of obstetric fistula in rural Tanzania	In-depth interview and informant interview	*n* = 25 median age = 35 years median age at fistula = 22	fear of fistula in future pregnancies
Janet MT, Khaliah J, and Mary LP, 2007	Eritrea, facility	Qualitative	Explore experiences of women seeking medical care for obstetric fistula in Eritrea: Implications for prevention, treatment, and social reintegration	In-depth interview	11 new patients, 15 women on pre repair follow-up and five accompanying family members mean age = 27 Years with fistula ranges 1–30 Duration of labour = 24 h-5 days	discomfort (such as soreness, irritation and itchiness in the genital area, and painful sexual intercourse); the need to wash constantly, being abandoned or divorced being unable to support themselves
Nsemo,2014	Nigeria, facility	Mixed design	Assess extent to which abandonment social isolation and stigma influence coping strategy	Structured and unstructured interview.	*n* = 120 patients, 18 follow ups,3 key informants, age12–30,duration of labor 6 h-4 days, duration of fistula 6 month-15 yrs	Divorce and rejection by spouse due to inactive sexual life, frustrated and withdrawn and resigned to fate... living on charity and begging.
Lilian and Thecla, Nov, 2015	Tanzania Rural, facility	Qualitative	To explore socio-cultural experiences of fistula patients	In-depth interview FGD	*n* = 28 (16 IDI,12 in FGD) mean age at interview = 29 year with fistula month- 19 years	Inability to work, being divorced, feel wet and smelly
Mselle, 2011	Tanzania(CCB R) Rural Facility + community	Quantitative and Qualitative (Mixed)	To explore physical cultural and psychosocial dimensions of living with obstetric fistula	Semi structured Interview Questionnaires FGD	*n* = 34(12 women with OF, 6 husband, 16 women on CBR) + 151 non fistula women. Age range 17–50 in qualitative 21-30 yrs. =39% < 18 year =18% Years with fistula = < 3 yrs. -6 yrs	Loss of body control, cleanness and social relations
Bangser et al., 2010	Tanzania and Uganda	Mixed	To explore women’s experience of their ‘near miss’ and experience of living with fistula	Semistructured interview and problem tree exercise Participatory approach,	*N* = 137 (Tanzania 61,Uganda 76) Duration with fistula: 1 month-52 years	Isolation, abandonment, lack of income, loss of hope to heal. Social stigma and sever economic hard ship are harsh
Okoye, et al., 2014	Nigeria facility, urban	Qualitative	Explore how women living with VVF cope with the health problem	In-depth interview	*n* = 10 fistula patients’ mean age 35 years, duration with fistula 10 - > 12 years, three married and two completed 12 year schooling.	Emotional trauma, physical and social challenges. Use of herbs to keep clean and sooth sores.
Gebresillasie, et al., 2014	Ethiopia, A.A (facility, urban)	Qualitative	To explore obstetric fistula survivors’ perception of social relation ship	In-depth interview. Ecological model	n = Eight women with OF selected purposively Mean age = 20–24 Years with fistula: 2–4 years, seven divorced and one separated.	pain, inability to eat as usual, emaciation spending most of the time lying on a bed and being dependent on people for simple things selling property, and orientation to reality and self isolation,
Kimani, 2014	Kenya, rural community	Mixed method	To evaluate the prevalence of obstetric fistula on women of Kaptembwa, and assess the impact on the well-being of women and how their experiences have shaped their life	Grounded theory for analysis	n = 120 respondents	Inability to keep clean and perform role, loss of dignity they miss out information on treatment and support due to lack of social interaction, could not involved in any economic activity and become dependent.
Kabayambi et al., 2014	Uganda urban, facility	Qualitative	To describe perceived causes, challenges faced and how women cope with the challenges	Semi structured interview KII with 10 Two FGD	*n* = 50 (30 women with fistula,10 care takers and 10 health care providers)	Constant wetness, loss of weight, inability to work, anxiety and depression, loss of baby Hiding from public, maintaining hygiene, drinking lot and eat less, ignoring comments, prayer.
Marissa, et al.., 2015	Malawi Rural, community	Qualitative	To gain an understanding of the lived experience of women obstetric fistula in Malawi.	In-depth interview	*n* = 45 women with fistula + 30 immediate family members Mean age of women 37 years.	Divorce, Feel useless, loss hope and thought of suicidal ideas
Proudence et al., 2013	Ghana, rural facility	Qualitative	To explores the experiences of Ghanaian women who sustained obstetric fistula during childbirth	In-depth interview	n = 10 women with obstetric fistula	Isolation, economical incapability lack of social interaction, could not involved in any economic activity and become dependent
Barageine, et al., 2015	Uganda Urban, facility	Qualitative	To explore the experiences of Ugandan women living with genital fistula	FGD	*n* = 56 women with obstetric fistula median age = 26 Duration with fistula: ranges between one and half month to forty years.	Consequences are Physical, social psychological, and Medical

The qualitative evidence was synthesized using thematic analysis. All themes identified in the primary studies were extracted. The thematic analysis allowed identification of major issues and helped to manage large data under each theme (Table 2). The steps were as follows:

1. Reading the document and identifying key issues and themes by relating to study objectives, contents of interview questions and respondents views and experiences;
2. Selecting similar themes from different studies in the same group and relabeling them with short descriptions;
3. Rearranging the data according to the appropriate part of the thematic framework to which they related, and
4. Using charts to define concepts, and to find associations between themes with a view to providing explanation of findings.

**Table 2:** Thematic excerpts and analysis of sixteen qualitative / mixed studies reporting challenges of obstetric fistula in sub Saharan Africa

Study	Sub theme	Excerpts	Main theme
Jenny. H and Sanna. S, 2014	1. Financial shortage 2. Challenged family life 3. Fertility 4. Social	“I want to see my children...never afford bus ticket. ‘He does not want to come closer or even to talk to me.’ ‘fear being pregnant’, I am not go out any more...”	1. Consequence on social and marital relationship 2. Economic incapability
Mselle et al., 2012	1. Emotional	“I am no longer observed as a wife”	1. Mental health consequences
Muleta et al., 2008	1. Social and marital relationship 2. ill health 3. difficulty of walking	“My husband’s relatives wanted him to divorce me, ‘for how long would you care for such a patient?’”	1. Physical consequence 2. Consequence on social and marital relationship
Pope, Bangser and Requejo, 2011	1. Emotional consequences 2. Not sure on recovery 3. Fear 4. Isolation	Fear of fistula in future pregnancy	1. Mental health consequence 2. Consequence on coping
Janet, Khaliah and Mary, 2007	1. Physical symptoms 2. Social isolation 3. Feel discomfort 4. Lose dignity	I cannot go out into the community. I have to wear a pad all the time...I don’t feel comfortable having sex.	1. Physical consequence 2. Consequence on social and marital relationship
Nsemo, 2014	1. Family life 2. Emotional 3. Financial	My husband understood that my situation is not improved, he rejected me...” They perceive fistula is \God’s curse “I believe my condition is will of God.” “a woman with fistula may beg for her survival” ...so I ask for money sitting at busy road and church corners...”	1. Consequence on social and marital relationship 2. Mental health consequences, 3. Economic incapability
Lilian and Thecla, 2015	1. Physical, 2. Social 3. Emotional	My life is in recession, I am not doing my business. I had a husband, but he left, ran away because of urine. I cannot sit with colleagues, ...I feel wet and smelly”	1. Consequence on social and marital relationship 2. Physical
Mselle et al., 2011	1. Loss of body control 2. Loss of social role as a woman and wife 3. Loss of integration 4. Loss of dignity and self worth	‘I always stay in my room due to strong smell from urine” “Now I could not return back to my husband because I am unable to perform my daily activities expected of me. I was staying at my mother’s house.’	1. Consequence on social and marital relationship
Bangser, 2010	1. Social stigma 2. Loss hope in recovery 3. Rejection 4. Food insecure/poverty	“... He left me and threw out all of my belongings” “Income has decreased ....there are times when we don’t have food” “I did not have hope that I would ever recover.’	1. Consequence on social and marital relationship 2. Economic incapability 3. Mental health consequences
Okoye, et al., 2014	1. Emotional 2. Hygiene 3. Pain, sores 4. Loss of hope 5. Use of herbs 6. Support seeking 7. Praying	“...they tell me the disease is reward from nature for all the evil things I did in my youth.” “The clothes get wet so easily the smell is humiliating with pains,...I get tired, this is not life” Attend religious crusades and visit pastors. They also come with money and gifts of wrappers and food “I use ‘powpow leaves water’ to bath and cool rashes and sores’	1. Mental health consequences 2. Physical consequence 3. Economic incapability 4. Consequences on coping
G/silasie et al., 2014	1. Powerlessness 2. Stigma 3. Divorce 4. Emotional challenges 5. Return to parents house	“...I went to my mother’s place when I am divorced since I had no choice”	1. Consequence on social and marital relationship 2. Consequences on coping
Kimani, 2014	1. Loss of body control 2. Inability to attend daily commitment 3. Loss of integration in social life 4. Loss of dignity and self worth.	“... my dresses are soaked with urine. ...it flows on its own my thighs irritated...” I could not perform day to day activities as a woman I only clean my belonging and bath rooms....” “... I lost contact with my friends, parents”. “is just like being in a status of child”	1. Physical consequence 2. Social consequences 3. Mental health consequences
Kabayambi et al., 2014	1. Physical 2. Psycho-social 3. Marital relationship, 4. Emotional/anxiety 5. Hiding from public 6. Drink lot and eat less	“my life has challenged and changed drastically...” “I am suffering from self pity, I cry a lot because I feel useless...”	1. Physical health consequence 2. Psychosocial challenge 3. Consequences on coping
Marissa, et al., 2009	1. Familial 2. Fertility and loss of pregnancy 3. Loss of association/integration	““... I just get pregnant but could not give live birth.” It is very sad for me to lose my husband because of something beyond my control” I lost interest in life and go to sleep frequently. ..I feel like that I should just die”	1. Psycho-social consequences 2. Marital relationship and fertility
Prudence, et al., 2013	1. Loss of integration with neighbors 2. Loss of job 3. Body sores 4. Constant wetness	“with soiled clothing I do not want to go to public it is embarrassing, so it is better to stay at home” “I used to sell cooked rice, My sales started dwindling as the news of my urine incontinence spread, Since then, I lost my job.” “I developed t skin sores at my under area very often. Sometimes the area becomes so severe itchy that I cannot able to bear it.”	1. Social consequences 2. Economical consequences 3. Physical consequences
Barageine, et al., 2009	1. Living a physically challenged life 2. Social deprivation 3. Self isolation 4. Stigma	“..both the young and the old share similar idea and keep saying that I smell when I cross them, people makes my life complicated. “... I feel like a burden to people. Neighbors and relatives don’t want to come to my place and I do the same. Because we fear each other” ... they were saying that my condition could never be treated, therefore I am bothered how to live with the problem the rest of my life..”	1. Psycho-social consequences 2. Physical consequences

## Results

**Fig. 1:**
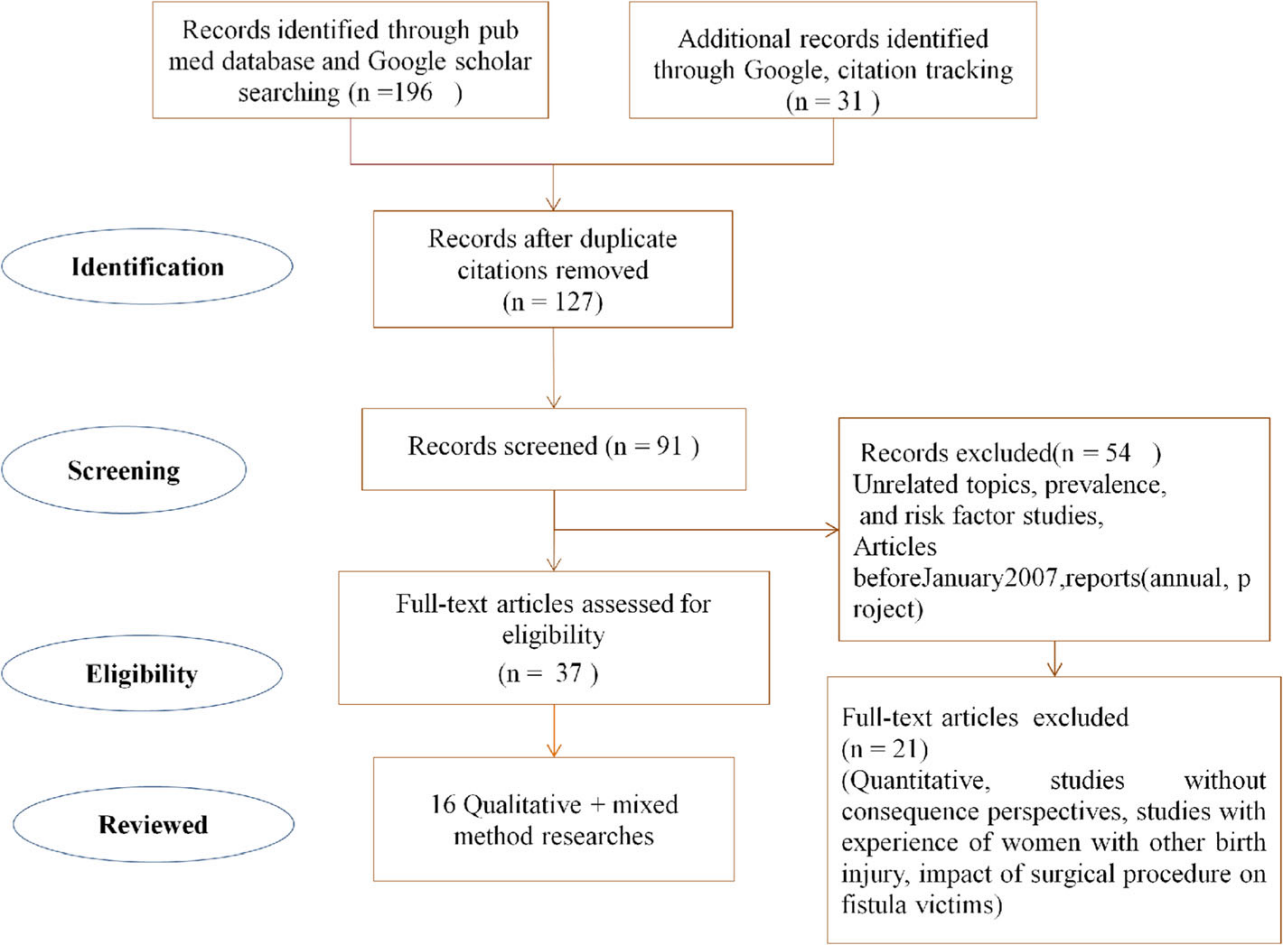
PRISMA flow diagram of included studies for systematic review of qualitative studies on consequences of Obstetric fistula in sub Sahara countries

A total of 127 citations were identified from the electronic search after removing duplicates. On review of the titles, 36 articles were discarded due to the irrelevance of the topic. On screening of the remaining 91 titles and abstracts, 54 were excluded based on the selection criteria. Nine were titles only and were not relevant to the objective of the review; and eight were annual or project reports. The remaining 37 studies were retrieved and read in full, of which 21 reports were excluded since they did not fulfill the eligibility criteria.19 articles were quantitative studies and two articles were qualitative but reported other than what was intended, i.e. one reported experiences of women with another birth injury, near miss uterine rupture; and the other reported the impact of surgical treatment on the mental health of fistula victims. The process used to determine which studies should be included in the review is shown in PRISMA flow diagram (Fig. 1).

All included studies were conducted in Sub-Saharan Africa: two in Ethiopia [21, 22], five in Tanzania [11, 13, 23–25], one in Uganda and Tanzania [11], one in Eritrea [16], two in Uganda [26, 27] two in Nigeria [19, 28], one in Kenya [29],one in Ghana [30], and one in Malawi [31]. All research took place in treatment or rehabilitation facilities, except reports from Kenya and Malawi which were conducted in the community. Nine studies were conducted in urban settings and the other seven in rural settings. Nine studies used a qualitative methods approach, while the rest used a mixed method approach. The stated length of study ranged from two weeks to two years between 2010 and 2013. All included studies related to the same target population, i.e. women affected by fistula, and two studies additionally included family members/care givers [13, 31]. Another two studies further incorporated key informants and experts in the field [11, 26]. Most studies used semi-structured interviews as a data collection tool, with the average study participants of 53 ranging from 8 to 137 respondents. The age of women included in the research ranged from 17 to 69 years. Nine studies reported the duration of fistula that ranged from one month to fifty two years.

The methodological quality of the included papers ranged from good to poor. The mean quality percentage score of the studies was 91%. Unreliable recruitment strategies, poor data collection methods, unclear researcher participant relationships and unclear data analysis methods were the limitations of the poor quality studies.

The thematic categories shared across the most studies were related to the physical challenges of losing body control, the disturbance in family relationship and women’s social association. Three studies included another theme, coping mechanism [22, 26, 32], and one study reported on mental health, marital relationships and child and fertility themes [31]. Some studies reported the loss of income source activities and medical conditions resulting from fistula (Table 3).

**Table 3:** Summary of Consequences of obstetric fistula in each of the included studies

Author(s) year of publication	Country	Focus of study (Objectives)	Design	Participants	Findings
Jenny. H and Sanna. S, 2014	Tanzania Facility	To learn about psychological consequences resulting from fistula.	NR (Mixed)	n = 63, mean age at fistula development = 31,mean time with fistula = 8.8 year	Decreased ability/ or inability to work, borrowed money, saved for bus fair for 3 years Divorced/rejected Lost babies and remain childless isolation
Mselle et al.,2012	Tanzania Facility	Explore Women’s experience of fistula	Qualitative	n = 8 median age = 30 yrs. median years with fistula = 6	Uncertainty about being accepted as a wife
Muleta et al., 2008	Ethiopia (Rural)	To assess health, social and psychological problems encountered by women	Mixed method	n = 13 median age = 33 age range at dev. of fistula = 15–49 median duration of labor = 4 days	amenorrhea, leg pain, difficulty of walking
Pope, Bangser and Requejo, 2011	Tanzania Facility	Explore barriers and facilitating factors women experience reintegrating in to society after treatment of obstetric fistula in rural Tanzania	Mixed methods	n = 25 median age = 35 years median age at fistula = 22	fear of fistula in future pregnancies
Janet, Khaliah and Mary, 2007	Eritrea, facility	Explore experiences of women seeking medical care for obstetric fistula in Eritrea: Implications for prevention, treatment, and social reintegration	Qualitative	11 new patients, 15 women on pre repair follow- up and five accompanying family members mean age = 27 Years with fistula ranges 1–30 Duration of labour = 24 h-5 days	discomfort (such as soreness, irritation and itchiness in the genital area, and painful sexual intercourse); the need to wash constantly, being abandoned or divorced being unable to support themselves
Nsemo, 2014	Nigeria, facility	Assess extent to which abandonment social isolation and stigma influence coping strategy	Mixed design	n = 120 patients,18 follow ups,3 key informants, age12–30,duration of labor 6 h- 4 days, duration of fistula 6 month-15 yrs	Divorce and rejection by spouse due to inactive sexual life, frustrated and withdrawn and resigned to fate... living on charity and begging.
Lilian and Thecla, 2015	Tanzania	To explore social-cultural experiences of women living with obstetric fistula in rural Tanzania	Qualitative	28 women with OF at Comprehensive Community Based Rehabilitation in Tanzania (CCBRT) (16for IDI,12 in FGD)	Social discrimination and loss of control, unable to work and contribute to the family income. Cannot satisfy their husband’s sexual needs
Mselle et al., 2011	Tanzania and Uganda	To explore physical cultural and psychosocial dimensions of living with obstetric fistula	Mixed (Mixed)	*n* = 185 (16 women at CCBR, 12 fistula patients, 151 other women, 6 husbands of fistula victims.	Deep sense of loss, loss of body control, loss of social role as woman and wife, loss of dignity and self worth
Bangser, 2010	Tanzania and	To explore women’s experience of near miss and experiences of living with fistula	Mixed	n = 137 (Tanzania 61,Uganda 76) and 391 through participatory approach	Social stigma and sever economic hard ship are the harsh consequences
Okoye, et al., 2014	Nigeria	Explore how women living with VVF experience and cope with the health problem	Qualitative	Ten women awaiting repairs at the National Fistula Centre at Abakaliki	Grief over the loss of their baby, but also the social repercussions that follow, often ostracized by their husbands, families and communities. Coping methods Bathing regularly and use of strips of old wrappers as pads.
G/silasie et al.2014	Ethiopia,	To explore OF survivors’ perception of social relation ship	Qualitative	Eight women with OF selected purposively	Powerlessness, physical injury, emotional breakdown, depression, erosion of social capital
Kimani, 2014	Kenya,	To evaluate the prevalence of obstetric fistula on women of Kaptembwa, and appraise the impact on the well-being of women and how their experiences have shaped their identities and families	Mixed	n = 120 respondents (women with obstetric fistula by snowball sampling and key informants)	Loss of integration in social life Loss of dignity and self worth Obstetric fistula has far reaching effects on affected women and their families (parental and marital) causing physical, social, economic and psychological impact.
Kabayambi et al., 2014	Uganda	To describe perceived causes, challenges faced and how women cope with the challenges	Qualitative	Median age at interview 27 years, Duration with OF ranges between two to 40 years.	physical, emotional, social, economic and spiritual aspects. The majority of the women had lost their babies and some their marriages. Coping: women with OF tended to cope through non-effective social measures including hiding from the general public.
Prudence, et al., 2013	Ghana	To explores the experiences of Ghanaian women who sustained obstetric fistula during childbirth	Qualitative	Age range 20–60 Period with fistula four to ten years.	Stigma and social isolation, worry about and coping with the odor, the emotional pain of stigmatization and various losses, marital disruption, and limited social support. Genital sores and rashes, intermittent abdominal pain, urinary tract infections, and menstrual changes
Marissa, et al., 2009	Malawi	To gain an understanding of the lived experience of obstetric fistula among Malawian women	Qualitative	n = 45 women with fistula + 30 immediate family members.	Divorce and marriage: in some cases woman’s relative encouraged the husband to take a second wife due to the woman’s condition to preserve the husband’s financial support.
Barageine, et al., 2009	Uganda	To explore the experiences of Ugandan women living with genital fistula	Qualitative	n = 56 women with obstetric fistula	Living a physically changed and challenging life, living in social deprivation and isolation, living psychologically stigmatized and depressed and living a marital and sexual life that is no longer joyful.

## Discussion

The review o demonstrated that the consequences of obstetric fistula are beyond its visible medical conditions in sub-Saharan Africa. This offers summarized evidences on consequences related to physical conditions; women’s social and marital relationships, economic in capabilities; mental health and challenges to coping mechanisms.

### Physical consequence

Physical consequence was the core theme shared across most studies, described as loss of body control and the challenges faced to keep cleanness [13, 16, 19, 24, 28, 29, 32]. Physical challenges include the emotional and medical problems of fistula which resulted from incontinence, such as genital sores, smells, wounds, pain, discomfort, exhaustion, and foot drop [21]. studies indicated that women with the condition were unable to afford hygiene keeping supplies and to cover medical fees therefore, they use herbs to sooth sores [11, 23]. In order to control the leak some women restrict themselves from feeding as they used to in pre fistula period, and this resulted in weight loss [11]. Moreover, in order to prevent the smell, discomfort, and to stay clean women with obstetric fistula tend to bath repeatedly; and they get bored of the frequency of washing [13, 32].

### Consequences on women’s social and marital relation ship

Most studies included marital and social relationships issues as the main theme [11, 13, 19, 22, 23, 26, 29]. It is a common event that fistula-affected women face divorce as they fail to satisfy their husband’s sexual needs and/or fail to bear children. As the women become incapable of performing the family roles expected of them, they were perceived as “useless” beings. Therefore, they became neglected and abandoned. A woman reported that she was mistreated by her spouse after the fistula incident, “He left me and threw out all of my belongings” …..ever since I developed the condition, we have not been together as husband and wife. I have been left out here to care for the old lady (mother-in-law). Even after repair, different socio-cultural factors can hinder the acceptability of women who integrated back to the community [13, 26, 31]. A study in Malawi reported that remarriage was not difficult among divorced women with fistula, as long as the husband knows the problem prior to marriage [31]. However, the report might not represent all other women with fistula, as it was reported by one participant and also may not be true for all fistula women living across different countries. Except for this report from Malawi, the concept of keeping association was also a common theme of all reviewed papers. Seven (43%) reported the faced discrimination and difficulty in maintaining social relationships. Unless they got an opportunity to obtain a repair service, women with fistula would not be considered as a woman and therefore, they lose their power and confidence [11]. Although few reported the presence of supportive husbands, most studies revealed experience of rejection, neglect and abandonment [8, 23, 24, 27, 31].

### Economic incapability

Loss of income source activities as a result of fistula was reported in some of the reviewed studies [22, 26, 27, 32]. Women with fistula could not get involved in gainful employment or activities that needed strict hygiene [32]. They also lost business because of the incontinence, and were self-employed in petty trading where they earned too little income. As a respondent from a Ghana study stated [32]: “*… I used to sell cooked rice but my condition has obliged me to stop. My sales started dwindling as the news of my urine incontinence was heard*”. Therefore, sometimes they fell into deeper poverty and start to beg for survival [32]. In this way, fistula has challenged and contributed to disfigure image of women’s health in developing countries.

### Mental health consequences

This issue was addressed in few of the studies that reported loss of dignity, lack of support, and lack of power to seek care. Loss of hope, fear of future life, and feelings of dependency were stated as mental health problems [13, 22]. This challenges were emerged as the result of different interrelated problems such as lack of support and family care, physical or economical incapability to access care, and lack of information or knowledge about fistula care and treatment [28]. It might also result from the reactions and comments of people with a poor understanding of the condition [19, 29]. Perceived causes of fistula and social stigma has caused psychological morbidity to women [19, 23, 27].

### Coping experiences

Keeping strict hygiene, washing with scented soap, the use of pieces of old cloth as a sanitary pad, and the use of herbs for cleaning, were reported as common coping mechanisms [22, 26, 32]. In some cases, ineffective practices for coping such as isolation, hiding, drinking a lot and eating less or changing feeding habits were undertaken [19]. Which may result in to other health problems such as depression and malnutrition [23, 27]. Women were used to discuss among themselves as a means for getting relief and those who got training tended to accept the reality in order to cope in a better way and keep themselves busy doing hand crafts [22].

Limited geographical coverage of the reviewed literatures might affect the transferability of the findings. Most of the included researches were conducted in facility settings, which mean that the voice of those women remaining at home, with perhaps more challenges, was lacking. The search strategy has not included subscription databases such as MEDLINE, EMBASE, and Scopus.

## Conclusion

This review highlighted that the consequences of obstetric fistula are far more than the visible medical condition itself. Little evidence is available on mental health, child and fertility issues, and coping mechanisms. Therefore, further research shall be aimed at addressing the understudied area, and suitable interventions shall be offered to improve women’s overall quality of life.

## Additional file

Additional file 1: Appraisal tool adopted from CASP used to assess quality of studies included in the review. (DOCX 49 kb)

## Abbreviations

CASP: Critical Appraisal Skills Program
CoCoPopS: Condition Context Population Study design
PRISMA: Preferred Reporting Items for Systematic review and Meta-Analyses
RVF: RectoVaginal Fistula
VVF: VesicoVaginal Fistula
WHO: World Health Organization
