# Supplementary material for: Consequences of obstetric fistula in sub Sahara African countries, from patients’ perspective: a systematic review of qualitative studies
Source: BMC Womens Health. 2018 Jun 20;18:106. doi: 10.1186/s12905-018-0605-1 (PMC6011512; doi:10.1186/s12905-018-0605-1)
Supplement: Supplementary file 1 — Appraisal tool adopted from CASP used to assess quality of studies included in the review. (DOCX 49 kb) [file 12905_2018_605_MOESM1_ESM.docx]

# Annex 1: Appraisal tool adopted from CASP quality assessment

|  | Criterion /evaluation questions | Points Considered |
| --- | --- | --- |
| 1 | Was there Clear statement of aims? | - What was the goal of the research? - Why it was thought important? - Is this relevant? |
| 2 | Was the qualitative method appropriate? | - Can it interpret or illuminate the actions and/or subjective experiences of research participants? - Is qualitative method the right methodology for addressing the research objectives? |
| 3 | Was the Study context Clearly described? | - Has the researcher justified the research design - Is the study characteristics (eg. location, setting) were easily identifiable? |
| 4 | Was the recruitment strategy appropriate to the aims of the research? | - If researcher explained how the participants were selected - If author explained why the participants selected were the most appropriate - If there are any discussions around recruitment |
| 5 | Were the data collected in a way that addresses the research issue? | - If the setting for data collection was justified - If it is clear how data were collected (e.g. focus group, semi structured interview etc.) - If the researcher has justified the methods chosen - If the researcher has made the methods explicit (e.g. for interview method, is there an indication of how interviews were conducted, or did they use a topic guide)? - If methods were modified during the study. If so, explained how and why - If the form of data is clear (e.g. tape recordings, video material, notes etc - If saturation of data discussed |
| 6 | Has the relationship between researcher and participant been considered and was there evidence of researcher reflexivity? | - If critically examined their own role, potential bias and influence during   - Formulation of the research questions   - Data collection, including sample recruitment and choice of location - How the researcher responded to events during the study and whether they considered the implication of any changes in the research design. |
| 7 | Have ethical issues been taken in to consideration? | - If there are sufficient details of how the research was explained - whether ethical standards were maintained - If the researcher has discussed issues raised by the study (e.g. issues around informed consent or confidentiality - how they have handled the effects of the study on the participants (during and after the study) - If approval has been sought from the ethics committee |
| 8 | Was the data analysis sufficiently rigorous? | - If there is an in depth description of the analysis process - If thematic analysis is used, If so, is it clear how the categories/themes were derived from the data - Whether the researcher explains how the data presented were selected from the original sample to demonstrate the analysis process - If sufficient data are presented to support the findings - To what extent contradictory data are taken into account - Whether critically examined their own role potential bias and influence during analysis and selection of data for presentation |
| 9 | Clear statement of findings | - If the findings are explicit - -If there is adequate discussion of the evidence both for and against the researchers arguments - -If the researcher has discussed the credibility of their findings (e.g. triangulation, respondent validation more than one analyst). If the findings are discussed in relation to the original research question |
| 10 | How valuable is the study? | - If the researcher discusses the contribution the study makes to existing knowledge or understanding e.g. in relation to current practice or policy? Or relevant research based literature? - If they identify new areas where research is necessary - If the researchers have discussed whether or how the findings can be transferred to other populations or considered other ways the research may be used |
